# Differences in Comorbidities and Clinical Burden of Severe Bronchopulmonary Dysplasia Based on Disease Severity

**DOI:** 10.3389/fped.2021.664033

**Published:** 2021-07-02

**Authors:** Hye-Rim Kim, Young Hwa Jung, Beyong Il Kim, So Young Kim, Chang Won Choi

**Affiliations:** ^1^Department of Pediatrics, Bundang CHA Medical Center, CHA University, Seongnam, South Korea; ^2^Department of Pediatrics, Seoul National University College of Medicine, Seoul, South Korea; ^3^Department of Pediatrics, Seoul National University Bundang Hospital, Seongnam, South Korea; ^4^Department of Otorhinolaryngology-Head & Neck Surgery, Bundang CHA Medical Center, CHA University, Seongnam, South Korea

**Keywords:** pre-term infants, very low birth weight, severe bronchopulmonary dysplasia, comorbidities of pre-maturity, neonatology

## Abstract

**Background:** The present study compared baseline characteristics, comorbidities and clinical burden of pre-term infants with type 1 and 2 severe bronchopulmonary dysplasia (BPD) Collaborative classification.

**Methods:** This study was a prospective cohort study of pre-term (<32 weeks) very-low-birth-weight infants. Severe BPD was divided into type 1 severe BPD requiring of ≥30% oxygen and/or non-invasive ventilation at 36 weeks post-menstrual age (PMA), and type 2 severe BPD requiring invasive mechanical ventilation at 36 weeks PMA. Baseline characteristics, comorbidities, and clinical burden were compared between these two types of severe BPD.

**Results:** Of the 1,328 infants included, 983 (74.0%) developed type 1 severe BPD, and 345 (26.0%) developed type 2 severe BPD. Lower birth weight, small for gestational age, lesser maternal pre-mature rupture of membrane, lower 5-min Apgar score, air leak, pulmonary hemorrhage, surgical ligation of patent ductus arteriosus, necrotizing enterocolitis, and late-onset sepsis were significantly associated with type 2 severe BPD. Compared with infants with type 1 severe BPD, infants with type 2 severe BPD had an increased risk of mortality (aOR 18.64, 95% CI 10.81–32.13), pulmonary hypertension (aOR 2.16, 95% CI 1.59–2.93), and tracheostomy (aOR 10.38, 95% CI 2.05–52.49).

**Conclusions:** Our data highlight the substantially greater mortality and clinical burden in infants with type 2 severe BPD than infants with type 1 severe BPD. A comprehensive and multidisciplinary approach is needed for infants with type 2 severe BPD.

## Introduction

Bronchopulmonary dysplasia (BPD) is one of the most common chronic morbidities in pre-term infants (1). BPD is commonly defined based on consensus recommendations from a National Institutes of Health (NIH) workshop (2). These recommendations classified BPD in pre-term infants born at <32 weeks as mild, moderate, or severe, according to the amount of supplemental oxygen and the mode of respiratory support administered at 36 weeks post-menstrual age (PMA).

Infants with severe BPD have higher mortality and morbidity than infants with mild or moderate BPD (3). Infants with severe BPD have complicated clinical courses and suffer substantial life-long burdens of pulmonary and neurodevelopmental sequelae (4–8). However, large variation in the severity of the disease is noted even within the severe BPD.

To better define BPD severity, a multi-institution group dedicated to filling knowledge gaps in the care of infants with BPD called the BPD Collaborative recommended that severe BPD be further classified into two phenotypes (9). Infants receiving persistent oxygen and/or nasal continuous positive airway pressure (CPAP) or high-flow nasal cannula (HFNC) at 36 weeks PMA were defined as having type 1 severe BPD, and infants requiring invasive mechanical ventilation were defined as having type 2 severe BPD. The BPD Collaborative reported a point prevalence of severe BPD of 36.5% in pre-term infants born at <32 weeks in tertiary referral neonatal intensive care units (NICUs), and 41% of these patients had type 2 severe BPD (10). Infants with type 2 severe BPD are more likely to die and have neurodevelopmental impairments (11).

However, the baseline characteristics and comorbidities associated with type 2 severe BPD remain understudied given its low incidence. Few reports have focused on perinatal factors that could affect disease severity and variation in the clinical burden within a severe BPD category.

In the present study, we identified the baseline characteristics associated with the development of type 2 severe BPD and assessed the clinical burden of type 2 severe BPD in compare to type 1 severe BPD using a large cohort of very-low-birth-weight (VLBW) infants.

## Materials and Methods

### Study Design

We performed a cohort study using prospectively collected data from 69 NICUs participating in the Korean Neonatal Network (KNN). The KNN is a nationwide multicenter registry of VLBW infants that collects demographic and clinical data using a standardized operating procedure (12).

**Figure 1:**
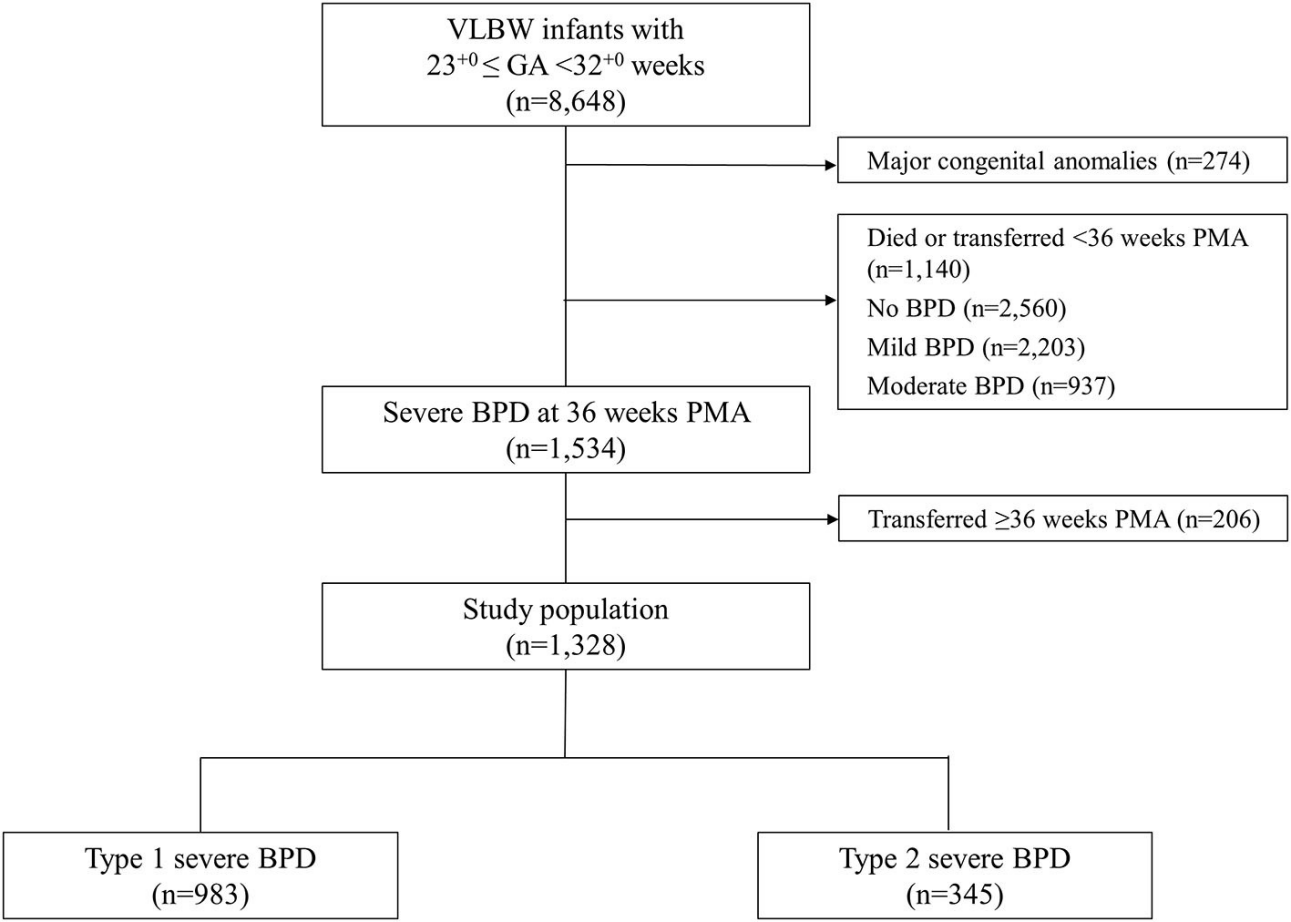
Study population. VLBW, very low birth weight; GA, gestational age; BPD, bronchopulmonary dysplasia; PMA, post-menstrual age.

Information on the study population is presented in Figure 1. We enrolled 8,648 VLBW infants registered in the KNN registry who were born at 23^+0^-31^+6^ gestational weeks between January 2013 and December 2017. Among these, 274 infants who had major congenital anomalies, 1,140 infants who died or were transferred to other hospitals before the diagnosis of BPD at 36 weeks PMA, and 2,560, 2,203, 937 infants with no, mild, moderate BPD, respectively were excluded. The remaining 1,534 infants had severe BPD, which was defined as the use of supplemental oxygen for at least 28 days plus treatment with ≥30% oxygen and/or positive pressure ventilation at 36 weeks PMA (2). Among these, 206 infants who were transferred to another hospital or ward in a hospital after 36 weeks PMA and for whom late morbidity data were unavailable were also excluded. Finally, data from 1,328 infants were analyzed. Infants with severe BPD were divided into infants with type 1 severe BPD and infants with type 2 severe BPD according to the BPD Collaborative recommendation (9). Infants with type 1 severe BPD were defined as requiring ≥30% oxygen and/or CPAP or HFNC at 36 weeks PMA, and infants with type 2 severe BPD were defined as requiring invasive mechanical ventilation at 36 weeks PMA.

### Data Collection

The maternal data included hypertension, diabetes, pre-mature rupture of membrane (PROM), histological chorioamnionitis, and the use of antenatal corticosteroids. The neonatal data included gestational age (GA), birth weight, sex, small for gestational age (SGA), multiple births, delivery mode, and Apgar scores at 1 and 5 min. The following clinical information was collected: respiratory distress syndrome (RDS), air leak, pulmonary hemorrhage, surgical ligation of patent ductus arteriosus (PDA), intraventricular hemorrhage (IVH), necrotizing enterocolitis (NEC), late-onset sepsis (LOS), postnatal corticosteroids, pulmonary hypertension, periventricular leukomalacia (PVL), retinopathy of pre-maturity (ROP) requiring treatment, the results of automated auditory brainstem response (AABR) before discharge, duration of invasive mechanical ventilation and non-invasive ventilation, length of NICU admission, and survival to NICU discharge or death. Because the KNN VLBW infant registry only collects data up to 12 months after birth, discharge information for infants who stayed in the NICU beyond 12 months of age were not available and not included in the analysis of discharge information. For surviving infants discharged from the NICU, we collected data on z-scores for weight, height, and head circumference at the time of discharge using Fenton 2013 growth curves (13).

The primary outcome was mortality after 36 weeks PMA during the NICU admission. Secondary outcomes were various neonatal comorbidities and clinical burden, including pulmonary hypertension, PVL, ROP requiring treatment, abnormal AABR results, duration of invasive mechanical ventilation and non-invasive ventilation, length of NICU admission, tracheostomy, the use of supplemental oxygen, and mechanical ventilation at NICU discharge.

### Definitions

Maternal hypertension included pre-existing hypertension and/or pregnancy-induced hypertension (14). Antenatal corticosteroid administration was defined as the successful completion of a dexamethasone or betamethasone regimen within 7 days before delivery. SGA was defined as birth weight below the 10th percentile for GA based on Fenton 2013 growth charts (13). RDS was defined by the presence of respiratory insufficiency that manifested at or shortly after birth with a typical radiological finding and required surfactant replacement therapy. NEC was defined as stage 2 or higher NEC according to modified Bell's staging criteria (15). High-grade IVH was defined as grade III or IV IVH according to the criteria of Papile classification system, and the worst grading result during hospitalization was recorded (16). LOS was defined as a culture-proven sepsis treated with appropriate antibiotics for 5 or more days which occurred after 72 h of life and prior to 36 weeks PMA (17). Pulmonary hypertension was diagnosed based on echocardiography and whether it required medical management. PVL was diagnosed based on the results of brain ultrasound or magnetic resonance imaging (MRI) findings before discharge. Invasive mechanical ventilation included conventional mechanical ventilation and high-frequency oscillatory ventilation. Non-invasive ventilation included nasal CPAP, nasal intermittent positive pressure ventilation, and HFNC.

### Statistical Analysis

Continuous variables are expressed as the means ± standard deviations; categorical variables are expressed as numbers and proportions. Comparisons of continuous variables between the groups were performed using Student's *t*-test for normally distributed variables and a *Mann-Whitney U*-test for variables with non-normal distributions. Categorical variables were compared using Pearson's chi-squared test. Multivariate analysis was performed using logistic regression, and adjusted odds ratios (aORs) and 95% confidence intervals (CIs) were calculated. Statistical analyses were performed using SPSS version 24.0 (IBM Corp., Armonk NY, USA). A *P* < 0.05 was considered statistically significant.

### Ethical Approval and Informed Consent

The registration of data in the KNN was approved by the institutional review board of each participating center. Informed consent was obtained from the parents of each infant prior to participation in the KNN registry.

## Results

The final study population included 1,328 pre-term (23^+0^-31^+6^ weeks) VLBW infants. Their mean GA was 27^+0^ ± 2^+0^ weeks, and their mean birth weight was 893 ± 253 g. Of these 1,328 infants, 983 (74.0%) infants developed type 1 severe BPD, and 345 (26.0%) infants developed type 2 severe BPD.

### Baseline Characteristics

The baseline characteristics are presented in Table 1. There were no significant differences in maternal characteristics between the type 1 and type 2 severe BPD groups, except maternal PROM, which was significantly more common in the type 1 severe BPD group than in the type 2 severe BPD group (41.0 vs. 34.4%, *p* = 0.038).

**Table 1:** Comparisons of baseline characteristics between the type 1 severe BPD and type 2 severe BPD groups.

	**Type 1 severe BPD**	**Type 2 severe BPD**	***p-*value**	Adjusted OR^*^	Adjusted *p-*value^*^
	***N* = 983**	***N* = 345**		**(95% CI)**	
Gestational age (weeks^+days^)	27^+1^ ± 2^+0^	26^+6^ ± 2^+0^	0.053		–
Birth weight (g)	909 ± 251	844 ± 253	<0.001		–
Male (%)	530 (53.9)	185 (53.6)	0.950		–
SGA (%)	144 (14.6)	83 (24.1)	<0.001		–
Cesarean section (%)	754 (76.7)	273 (79.1)	0.371		–
Multiple gestation (%)	302 (30.7)	97 (28.1)	0.376		–
Maternal hypertension (%)	192 (19.5)	62 (18.0)	0.578		–
Maternal diabetes (%)	79 (8.0)	24 (7.0)	0.560		–
Maternal PROM (%)^**^	400 (41.0)	116 (34.4)	0.038		–
Histologic chorioamnionitis (%)^†^	388 (45.8)	113 (42.2)	0.324		–
Antenatal corticosteroids (%)	490 (49.8)	172 (49.9)	0.524		–
1-min Apgar score	4.0 ± 1.8	3.7 ± 1.8	0.014	0.943 (0.876–1.015)	0.115
5-min Apgar score	6.3 ± 1.8	6.0 ± 1.8	0.006	0.931 (0.867–0.999)	0.048
Respiratory distress syndrome (%)	929 (94.5)	334 (96.8)	0.110	1.582 (0.802–3.122)	0.186
Air leak (%)	67 (6.8)	48 (13.9)	<0.001	2.029 (1.350–3.049)	0.001
Pulmonary hemorrhage (%)	77 (7.8)	62 (18.0)	<0.001	2.493 (1.725–3.603)	<0.001
Surgical ligation of PDA (%)	239 (24.3)	133 (38.6)	<0.001	1.872 (1.427–2.455)	<0.001
High-grade IVH	215 (21.9)	93 (27.0)	0.064	1.290 (0.963–1.728)	0.088
NEC (≥stage II)	99 (10.1)	62 (18.0)	<0.001	1.808 (1.267–2.580)	0.001
LOS before 36 weeks PMA (%)	294 (29.9)	139 (40.3)	0.001	1.535 (1.182–1.994)	0.001

*Values are presented as means ± SDs or numbers (%). BPD, bronchopulmonary dysplasia; OR, odds ratio; CI, confidence interval; SGA, small for gestational age; PROM, pre-mature rupture of membrane; PDA, patent ductus arteriosus; IVH, intraventricular hemorrhage; NEC, necrotizing enterocolitis; LOS, late-onset sepsis; PMA, post-menstrual age*.

* *The ORs and p-values were calculated with adjustment for gestational age, birth weight, sex, SGA and maternal pre-mature rupture of membrane*.

** *Data were available for 976 infants in the type 1 severe BPD group and 337 infants in the type 2 severe BPD group*.

† *Data were available for 847 infants in the type 1 severe BPD group and 268 infants in the type 2 severe BPD group*.

Compared to infants with type 1 severe BPD, infants with type 2 severe BPD had a lower birth weight, lower 5-min Apgar score and a higher rate of SGA. Infants who developed type 2 severe BPD also had significantly higher rates of air leak, pulmonary hemorrhage, surgical ligation of PDA, NEC, and LOS (Table 1).

### Mortality, Comorbidities, and Clinical Burden

**Figure 2:**
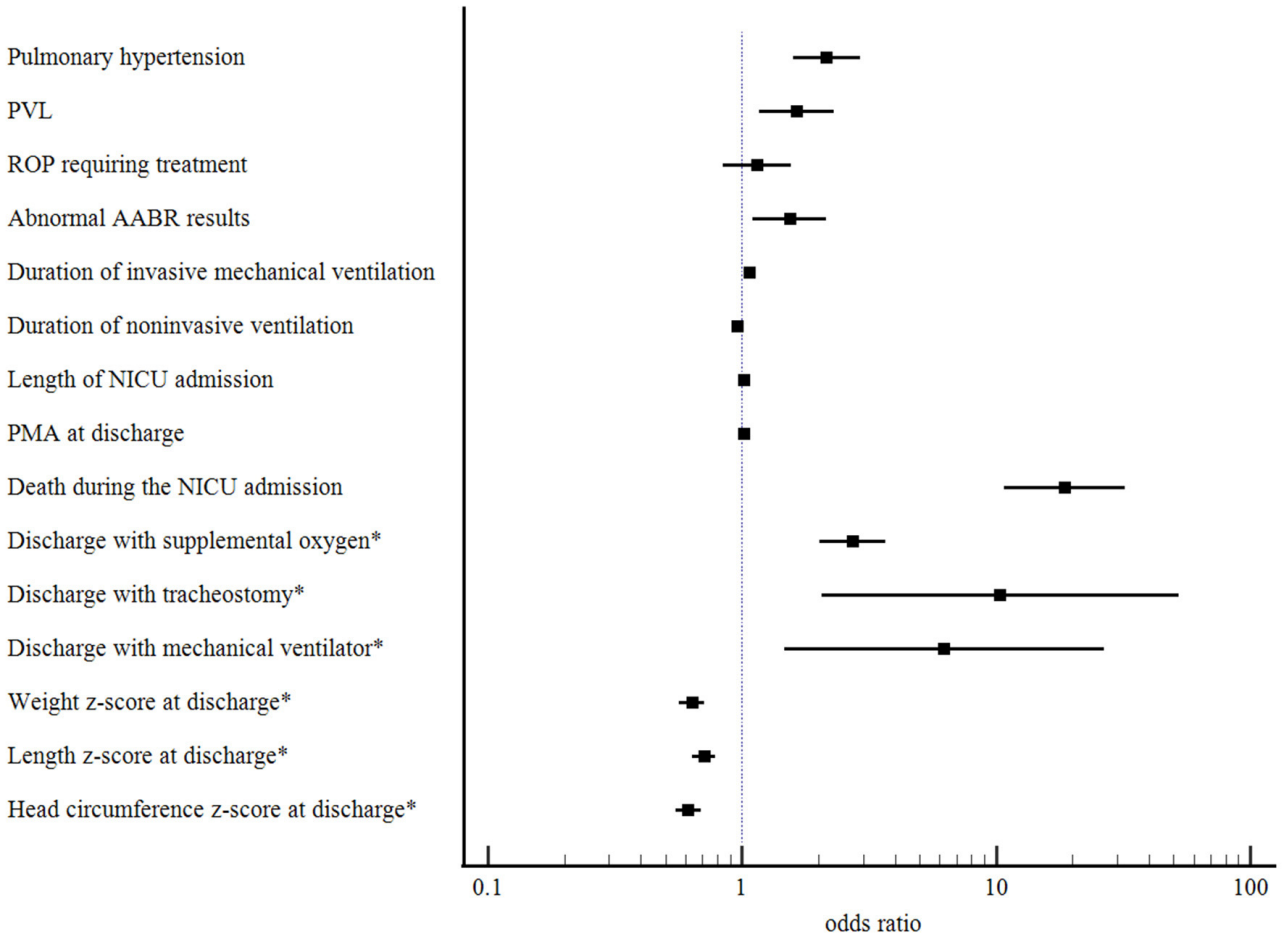
Adjusted odds ratios with their 95% confidence intervals for comorbidities and clinical burden of the type 2 severe BPD compared to those of the type 1 severe BPD. BPD, bronchopulmonary dysplasia; PVL, periventricular leukomalacia; ROP, retinopathy of pre-maturity; AABR, automated auditory brainstem response; NICU, neonatal intensive care unit; PMA, post-menstrual age. *Calculated for survivors to NICU discharge.

The mortality, comorbidities, and clinical burden of the type 2 severe BPD group were compared to those of the type 1 severe BPD group (Table 2). Overall, 8.2% (*n* = 109) of the infants with severe BPD died before discharge, and 0.5% (*n* = 7) remained in the NICU beyond 12 months of age. Cardiorespiratory failure was the most common cause of death and accounted for 40% of all deaths. The mortality rate during the NICU admission was 1.9% in the type 1 severe BPD group and 26.1% in the type 2 severe BPD group (Table 2). After adjustment for GA, birth weight, sex, SGA and maternal PROM, the aOR for death during the NICU admission increased to 18.64 (95% CI 10.81–32.13, *p* < 0.001) for infants with type 2 severe BPD compared to infants with type 1 severe BPD (Table 3, Figure 2).

**Table 2:** Comparisons of comorbidities and clinical burden between the type 1 and type 2 severe BPD groups.

	**Type 1 severe BPD**	**Type 2 severe BPD**	***p-*value**
	***N* = 983**	***N* = 345**	
Postnatal corticosteroids (%)	580 (59.0)	256 (74.2)	<0.001
Pulmonary hypertension (%)	152 (15.5)	102 (29.6)	<0.001
PVL (%)	125 (12.7)	66 (19.1)	0.004
ROP requiring treatment (%)	292 (29.7)	128 (37.4)	0.013
Abnormal AABR results (%)^*^	190 (20.9)	68 (30.1)	0.004
Duration of invasive mechanical ventilation (days)	32.0 ± 27.7	83.8 ± 51.1	<0.001
Duration of non-invasive ventilation (days)	44.8 ± 24.4	26.9 ± 31.7	<0.001
Length of NICU admission (days)	109.3 ± 38.7	137.3 ± 59.0	<0.001
PMA at discharge (weeks)	42^+4^ ± 4^+4^	46^+3^ ± 8^+0^	<0.001
Death during the NICU admission (%)	19 (1.9)	90 (26.1)	<0.001
Discharge with supplemental oxygen (%)^**^	241 (25.1)	120 (47.6)	<0.001
Discharge with tracheostomy (%)^**^	2 (0.2)	6 (2.4)	0.001
Discharge with mechanical ventilator (%)^**^	3 (0.3)	5 (2.0)	0.012
Weight z-score at discharge^**^	−1.74 ± 1.45	−2.59 ± 1.66	<0.001
Length z-score at discharge^**^	−2.44 ± 1.71	−3.40 ± 2.16	<0.001
Head circumference z-score at discharge^**^	−1.47 ± 1.38	−2.60 ± 2.14	<0.001

*Values are presented as means ± SDs or numbers (%). BPD, bronchopulmonary dysplasia; PVL, periventricular leukomalacia; ROP, retinopathy of pre-maturity; AABR, automated auditory brainstem response; NICU, neonatal intensive care unit; PMA, post-menstrual age*.

* *Data were available for 909 infants in the type 1 severe BPD group and 225 infants in the type 2 severe BPD group*.

** *Calculated for survivors to NICU discharge*.

**Table 3:** Adjusted odds ratios and *p*-values for comorbidities and clinical burden of type 2 severe BPD compared to those of type 1 severe BPD.

	Adjusted OR^*^	Adjusted *p-*value^*^
	**(95% CI)**	
Pulmonary hypertension	2.155 (1.587–2.927)	<0.001
PVL	1.636 (1.168–2.292)	0.004
ROP requiring treatment	1.144 (0.840–1.558)	0.392
Abnormal AABR results	1.538 (1.102–2.148)	0.011
Duration of invasive mechanical ventilation	1.065 (1.057–1.073)	<0.001
Duration of non-invasive ventilation	0.962 (0.955–0.969)	<0.001
Length of NICU admission	1.014 (1.010–1.017)	<0.001
PMA at discharge	1.014 (1.010–1.017)	<0.001
Death during the NICU admission	18.635 (10.807–32.132)	<0.001
Discharge with supplemental oxygen^**^	2.730 (2.025–3.682)	<0.001
Discharge with tracheostomy^**^	10.380 (2.053–52.487)	0.005
Discharge with mechanical ventilator^**^	6.250 (1.467–26.639)	0.013
Weight z-score at discharge^**^	0.635 (0.566–0.712)	<0.001
Length z-score at discharge^**^	0.709 (0.640–0.787)	<0.001
Head circumference z-score at discharge^**^	0.615 (0.547–0.691)	<0.001

*BPD; bronchopulmonary dysplasia; OR, odds ratio; CI, confidence interval; PVL, periventricular leukomalacia; ROP, retinopathy of pre-maturity; AABR, automated auditory brainstem response; NICU, neonatal intensive care unit; PMA, post-menstrual age*.

* *The ORs and p-values were calculated using binary logistic regression analysis with adjustment for gestational age, birth weight, sex, small for gestational age, and maternal pre-mature rupture of membrane*.

** *Calculated for survivors to NICU discharge*.

The type 2 severe BPD group had significantly higher rates of comorbidities, including pulmonary hypertension, ROP requiring treatment, PVL, and abnormal AABR results, than the type 1 severe BPD group. Infants with type 2 severe BPD had a higher clinical burden as represented by a longer duration of invasive mechanical ventilation, and NICU admission than infants with type 1 severe BPD. The type 2 severe BPD group had lower z-scores for weight, height, and head circumference at the time of NICU discharge (Table 2).

After adjustment for GA, birth weight, sex, SGA, and maternal PROM, the odds of pulmonary hypertension increased significantly in in infants with type 2 severe BPD (aOR 2.16, 95% CI 1.59–2.93, *p* < 0.001). The odds of PVL (aOR 1.64, 95% CI 1.17–2.29, *p* = 0.004) and abnormal AABR results (aOR 1.54, 95% CI 1.10–2.15, *p* = 0.011) were also significantly increased in these infants. Regarding the clinical burden, the type 2 severe BPD group was associated with a longer duration of invasive mechanical ventilation and NICU admission, and increased risks of discharge with home oxygen (aOR 2.73, 95% CI 2.03–3.68, *p* < 0.001), tracheostomy (aOR 10.38, 95% CI 2.05–52.49, *p* = 0.005), and mechanical ventilation (aOR 6.25, 95% CI 1.47–26.64, *p* = 0.013). For somatic growth at the time of discharge, the type 2 severe BPD group was associated with lower z-scores for weight, height, and head circumference (Table 3, Figure 2).

## Discussion

Our results showed that infants with the type 2 severe BPD had an 18-fold increased risk of mortality, a 2-fold increased risk of f pulmonary hypertension, and a 10-fold increased risk of tracheostomy and a greater clinical burden as indicated by a longer duration of invasive mechanical ventilation and NICU admission than infants with the type 1 severe BPD. Among the baseline characteristics, lower birth weight, SGA, lesser maternal PROM, lower 5-min Apgar score, air leak, pulmonary hemorrhage, surgical ligation of PDA, NEC, and LOS were significantly associated with type 2 severe BPD. These antenatal and postnatal factors may predispose the immature lung to injuries or directly cause injury to the immature lung and contribute to impaired alveolar and pulmonary vascular development, which increases the risk of type 2 severe BPD (18). Other investigators noted similar observations that PDA, sepsis, and surgical NEC had a significant role in the development of severe BPD (19, 20). The presence of PDA has been associated with the development of BPD (21, 22). The persistence of left-to-right shunting through PDA results in pulmonary edema and endothelial injury. These alterations lead to the prolonged need for high levels of respiratory support, including invasive mechanical ventilation and high inspired oxygen concentrations. Surgical ligation of PDA may indicate the greater severity of PDA, which may lead to exacerbation of lung disease, such as type 2 severe BPD. However, in addition to being reflective of a significant left-to-right shunt, there is evidence that surgical ligation of PDA itself may contribute to lung injury directly (23). Sepsis is also related to the development of BPD (17). There is considerable evidence to support that postnatal infection exacerbates the severity of BPD (24, 25). Jung and Lee recently demonstrated that LOS was a risk factor for BPD in extremely low birth weight (ELBW) infants using the KNN database (26). Because we limited LOS to cases that occurred before 36 weeks PMA when the diagnosis of BPD was made, our results support the role of LOS as a risk factor for more severe type of BPD.

Severe BPD results in high mortality and extensive morbidities (3). More specifically, infants with severe BPD are at an increased risk of long-term serious morbidities, including late pulmonary morbidity, pulmonary hypertension, gastroesophageal reflux, feeding difficulty, ROP, systemic hypertension, and neurodevelopmental impairment (4–8). Infants with severe BPD receiving invasive mechanical ventilation at 36 weeks PMA also had an increased risk of late mortality, severe respiratory morbidities, poor growth, and neurodevelopmental impairment than infants receiving noninvasive ventilation at 36 weeks PMA (11). We found that infants with type 2 severe BPD had an 18-fold increased risk of death during the NICU admission compared to infants with type 1 severe BPD, and the mortality rate reached 26% in infants with type 2 severe BPD. Type 2 severe BPD was strongly associated with a longer duration of invasive mechanical ventilation. At NICU discharge, infants with type 2 severe BPD were associated with a 10-fold increase in tracheostomy, a 3-fold increase in supplemental oxygen use, and a 6-fold increase in mechanical ventilator requirement. These results suggest that infants with type 2 severe BPD are at a high risk of having chronic respiratory support and undergoing tracheostomy. These findings may reflect the disease severity and indicate that infants with type 2 severe BPD had more severe lung parenchymal and airway diseases. In our study population, the rates of tracheostomy and mechanical ventilation at discharge were 0.5 and 0.7%, respectively, which were much lower than those of previously reported referral-based cohort (27). According to a report from Children's Hospital Neonatal Consortium on infants with severe BPD born at <32 weeks, 5% had tracheostomy and 4% required mechanical ventilation at the time of discharge (27). The reason for this discrepancy may be due to the different study populations. Their study included pre-term infants who were admitted to tertiary referral hospitals. In contrast, we excluded from the analysis 206 infants who were transferred to another hospital or unit after 36 weeks PMA. These infants were often more ill than those who stayed at their initial hospital until NICU discharge. Of the 206 infants who were transferred, 121 infants had type 1 severe BPD and 85 infants had type 2 severe BPD. Because infants with type 2 severe BPD were transferred twice as many as infants with type 1 severe BPD after 36 weeks PMA, exclusion of these infants might have led to under-reporting of the actual rates of tracheostomy and mechanical ventilator use at discharge. Similarly, exclusion of these infants might have reduced the gaps in mortality, comorbidities, and clinical burden between type 1 and type 2 severe BPD.

We also found that infants with type 2 severe BPD were subject to significant comorbidities, including pulmonary hypertension, PVL, and impaired auditory response. Pulmonary hypertension developed in 20% of severe BPD infants, which is consistent with prior reports and highlights the importance of pulmonary hypertension as a comorbidity in infants with severe BPD (28, 29). After adjusting for confounding variables, the odds of developing pulmonary hypertension were increased 2-fold in infants with type 2 severe BPD compared with infants with type 1 severe BPD. A diagnosis of BPD-associated pulmonary hypertension was consistently associated with 2-year mortality rates as high as 40–50% (30, 31). Pulmonary hypertension has also been linked to an increased risk of death or tracheostomy (32, 33).

Infants with BPD also have difficulty maintaining growth (34). Based on the lower z-scores of weight, height, and head circumference at the time of discharge, we found that infants with type 2 severe BPD grew more poorly. The causes of growth failure in infants with type 2 severe BPD may include increased work of breathing, chronic stress and inflammation, and feeding problems associated with respiratory insufficiency.

The strength of our study is that we evaluated baseline characteristics, mortality, comorbidities, and clinical burden of severe BPD based on disease severity in a large, multicenter prospective cohort. However, there are several limitations that should be mentioned. First, we limited our analysis to infants who survived to 36 weeks PMA. Infants with the most severe BPD might have not survived to 36 weeks PMA. As many as half of all deaths in very pre-term infants before 36 weeks PMA are pulmonary-related (1, 35). This might have led to under-reporting of the disease severity of type 2 severe BPD. Secondly, as mentioned above, exclusion of 206 infants who were transferred after 36 weeks PMA due to unavailability of late morbidity data might have reduced the gaps in comorbidities and clinical burden between type 1 and type 2 severe BPD. Finally, we did not examine long-term outcome data beyond NICU discharge.

In conclusion, the present study identified baseline characteristics, comorbidities, and clinical burden associated with type 2 severe BPD with a cohort of pre-term VLBW infants. Type 2 severe BPD was associated with a substantially high rate of various comorbidities and an enormous clinical burden. Stratifying infants with severe BPD into subtypes based on disease severity will likely improve the care of infants with more severe BPD. Our data highlight the need for a better understanding of the specific etiologies of type 2 severe BPD and a comprehensive, multidisciplinary approach for infants with type 2 severe BPD.

## Data Availability Statement

The datasets presented in this article are not readily available because the KNN Publication Ethics Policy adheres to the following research data management and access guidelines: All information about patients and participating NICUs is confidential and is only available to individuals who have access for the purposes of the research activities permitted. Access is only allowed for the purpose of collecting data for the first time, and no access for any other purpose is allowed. Requests to access the datasets should be directed to Yun Sil Chang (yschang@skky.edu).

## Ethics Statement

The registration of data in the KNN was approved by the institutional review board of each participating center. Informed consent was obtained from the parents of each infant prior to participation in the KNN registry. Written informed consent to participate in this study was provided by the participants' legal guardian/next of kin.

## Author Contributions

H-RK and CC performed the research, analyzed the data, and wrote the manuscript. YJ and CC designed the study. CC reviewed and revised the manuscripts. BK and SK provided suggestions with regard to the content and concept of the manuscript. All authors read and approved the final manuscript.

## Conflict of Interest

The authors declare that the research was conducted in the absence of any commercial or financial relationships that could be construed as a potential conflict of interest.

## Abbreviations

AABR: automated auditory brainstem response
BPD: bronchopulmonary dysplasia
CI: confidence interval
CPAP: continuous positive airway pressure
ELBW: extremely low birth weight
EOS: early-onset sepsis
GA: gestational age
HFNC: high-flow nasal cannula
IVH: intraventricular hemorrhage
KNN: Korean Neonatal Network
LOS: late-onset sepsis
MRI: magnetic resonance imaging
NEC: necrotizing enterocolitis
NICU: neonatal intensive care unit
NIH: National Institutes of Health
OR: odds ratio
PDA: persistent ductus arteriosus
PMA: post-menstrual age
PROM: pre-mature rupture of membrane
PVL: periventricular leukomalacia
RDS: respiratory distress syndrome
ROP: retinopathy of pre-maturity
SGA: small for gestational age
VLBW: very low birth weight.

## References

[1] Poindexter BB Feng R Schmidt B Aschner J Ballard RA Hamvas A. Comparisons and limitations of current definitions of bronchopulmonary dysplasia for the prematurity and respiratory outcomes program. Ann Am Thorac. (2015) 12:1822–30. 10.1513/AnnalsATS.201504-218OC 26397992 PMC4722827

[2] Jobe AH Bancalari E. Bronchopulmonary dysplasia. Am J Respir Crit Care Med. (2001) 163:1723–9. 10.1164/ajrccm.163.7.2011060 11401896

[3] Ehrenkranz RA Walsh MC Vohr BR Jobe AH Wright LL Fanaroff AA. Validation of the national institutes of health consensus definition of bronchopulmonary dysplasia. Pediatrics. (2005) 116:1353–60. 10.1542/peds.2005-0249 16322158

[4] Cristea AI Carroll AE Davis SD Swigonski NL Ackerman VL. Outcomes of children with severe bronchopulmonary dysplasia who were ventilator dependent at home. Pediatrics. (2013) 132:e727–34. 10.1542/peds.2012-2990 23918888 PMC3876749

[5] Smith VC Zupancic JA McCormick MC Croen LA Creene J Escobar GJ. Rehospitalization in the first year of life among infants with bronchopulmonary dysplasia. J Pediatr. (2004) 144:799–803. 10.1016/j.jpeds.2004.03.026 15192629

[6] Schmidt B Asztalos EV Roberts RS Robertson CM Sauve RS Whitfield MF. Impact of bronchopulmonary dysplasia, brain injury, and severe retinopathy on the outcome of extremely low-birth-weight infants at 18 months: results from the trial of indomethacin prophylaxis in preterms. JAMA. (2003) 289:1124–9. 10.1001/jama.289.9.1124 12622582

[7] Anderson PJ Doyle LW. Neurodevelopmental outcome of bronchopulmonary dysplasia. Semin Perinatol. (2006) 30:227–32. 10.1053/j.semperi.2006.05.010 16860163

[8] Jeng SF Hsu CH Tsao PN Chou HC Lee WT Kao HA. Bronchopulmonary dysplasia predicts adverse developmental and clinical outcomes in very-low-birth-weight infants. Dev Med Child Neurol. (2008) 50:51–7. 10.1111/j.1469-8749.2007.02011.x 18173631

[9] Abman SH Collaco JM Shepherd EG Keszler M Cuevas-Guaman M Welty SE. Interdisciplinary care of children with severe bronchopulmonary dysplasia. J Pediatr. (2017) 181:12–28. 10.1016/j.jpeds.2016.10.082 27908648 PMC5562402

[10] Guaman MC Gien J Baker CD Zhang H Austin ED Collaco JM. Point prevalence, clinical characteristics, and treatment variation for infants with severe bronchopulmonary dysplasia. Am J Perinatol. (2015) 32:960–7. 10.1055/s-0035-1547326 25738785 PMC4617756

[11] Jensen EA Dysart K Gantz MG McDonald S Bamat NA Keszler M. The diagnosis of bronchopulmonary dysplasia in very preterm infants. An evidence-based approach. Am J Respir Crit Care Med. (2019) 200:751–9. 10.1164/rccm.201812-2348OC 30995069 PMC6775872

[12] Jang YS Park HY Park WS. The Korean neonatal network: an overview. J Korean Med Sci. (2015) 30:S3–11. 10.3346/jkms.2015.30.S1.S3 26566355 PMC4641061

[13] Fenton TR Kim JH. A systematic review and meta-analysis to revise the Fenton growth chart for preterm infants. BMC Pediatr. (2013) 13:59. 10.1186/1471-2431-13-59 23601190 PMC3637477

[14] American College of Obstetricians and Gynecologists Task Force on Hypertension in Pregnancy. Hypertension in pregnancy. Report of the American College of Obstetricians and Gynecologists' Task Force on Hypertension in Pregnancy. Obstet Gynecol. (2013) 122:1122–31. 10.1097/01.AOG.0000437382.03963.88 24150027

[15] Bell MJ Ternberg JL Feigin RD Keating JP Marshall R Barton L. Neonatal necrotizing enterocolitis. Therapeutic decisions based upon clinical staging. Ann Surg. (1978) 187:1–7. 10.1097/00000658-197801000-00001 413500 PMC1396409

[16] Papile LA Burstein J Burstein R Koffler H. Incidence and evolution of subependymal and intraventricular hemorrhage: a study of infants with birth weights less than 1,500 gm. J Pediatr. (1978) 92:529–34. 10.1016/S0022-3476(78)80282-0 305471

[17] Stoll BJ Hansen N Fanaroff AA Wright LL Carlo WA Ehrenkranz RA. Late-onset sepsis in very low birth weight neonates: the experience of the NICHD neonatal research network. Pediatrics. (2002) 110:285–91. 10.1542/peds.110.2.285 12165580

[18] Jain D Bancalari E. Bronchopulmonary dysplasia: clinical perspective. Birth Defects Res A Clin Mol Teratol. (2014) 100:134–44. 10.1002/bdra.23229 24578124

[19] Marshall DD Kotelchuck M Young TE Bose CL Kruyer L O'Shea TM. Risk factors for chronic lung disease in the surfactant era: a North Carolina population-based study of very low birth weight infants. North Carolina neonatologists association. Pediatrics. (1999) 104:1345–50. 10.1542/peds.104.6.1345 10585987

[20] Oh W Poindexter BB Perritt R Lemons JA Bauer CR Ehrenkranz RA. Neonatal Research Network. Association between fluid intake and weight loss during the first ten days of life and risk of bronchopulmonary dysplasia in extremely low birth weight infants. J Pediatr. (2005) 147:786–90. 10.1016/j.jpeds.2005.06.039 16356432

[21] Clyman RI. Patent ductus arteriosus, its treatments, and the risks of pulmonary morbidity. Semin Perinatol. (2018) 42:235–42. 10.1053/j.semperi.2018.05.006 29958703

[22] Schena F Francescato G Cappelleri A Picciolli I Mayer A Mosca F. Association between hemodynamically significant patent ductus arteriosus and bronchopulmonary dysplasia. J Pediatr. (2015) 166:1488–92. 10.1016/j.jpeds.2015.03.012 25882876

[23] Barikbin P Sallmon H Wilitzki S Photiadis J Bührer C Koehne P. Lung function in very low birth weight infants after pharmacological and surgical treatment of patent ductus arteriosus - a retrospective analysis. BMC Pediatr. (2017) 17:5. 10.1186/s12887-016-0762-z 28056907 PMC5217232

[24] Shah J Jefferies AL Yoon EW Lee SK Shah PS. Canadian Neonatal Network. Risk factors and outcomes of late-onset bacterial sepsis in preterm neonates born at <32 weeks' gestation. Am J Perinatol. (2015) 32:675–82. 10.1055/s-0034-1393936 25486288

[25] Landry JS Menzies D. Occurrence and severity of bronchopulmonary dysplasia and respiratory distress syndrome after a preterm birth. Paediatr Child Health. (2011) 16:399–403. 10.1093/pch/16.7.399 22851893 PMC3200385

[26] Jung E Lee BS. Late-onset sepsis as a risk factor for bronchopulmonary dysplasia in extremely low birth weight infants: a nationwide cohort study. Sci Rep. (2019) 9:15448. 10.1038/s41598-019-51617-8 31664055 PMC6820783

[27] Padula MA Grover TR Brozanski B Zaniletti I Nelin LD Asselin JM. Therapeutic interventions and short-term outcomes for infants with severe bronchopulmonary dysplasia born at <32 weeks' gestation. J Perinatol. (2013) 33:877–81. 10.1038/jp.2013.75 23828204

[28] Ali Z Schmidt P Dodd J Jeppesen DL. Predictors of bronchopulmonary dysplasia and pulmonary hypertension in newborn children. Dan Med J. (2013) 60:A4688. 23905570

[29] An HS Bae EJ Kim GB Kwon BS Beak JS Kim EK. Pulmonary hypertension in preterm infants with bronchopulmonary dysplasia. Korean Circ J. (2010) 40:131–6. 10.4070/kcj.2010.40.3.131 20339498 PMC2844979

[30] Khemani E McElhinney DB Rhein L Andrade O Lacro RV Thomas KC. Pulmonary artery hypertension in formerly premature infants with bronchopulmonary dysplasia: clinical features and outcomes in the surfactant era. Pediatrics. (2007) 120:1260–9. 10.1542/peds.2007-0971 18055675

[31] del Cerro MJ Sabaté Rotés A Cartón A Deiros L Bret M Cordeiro M. Pulmonary hypertension in bronchopulmonary dysplasia: clinical findings, cardiovascular anomalies and outcomes. Pediatr Pulmonol. (2014) 49:49–59. 10.1002/ppul.22797 23788443

[32] Bhat R Salas AA Foster C Carlo WA Ambalavanan N. Prospective analysis of pulmonary hypertension in extremely low birth weight infants. Pediatrics. (2012) 129:e682–9. 10.1542/peds.2011-1827 22311993 PMC3289526

[33] Murthy K Savani RC Lagatta JM Zaniletti I Wadhawan R Truog W. Predicting death or tracheostomy placement in infants with severe bronchopulmonary dysplasia. J Perinatol. (2014) 34:543–8. 10.1038/jp.2014.35 24651732

[34] Jensen EA Schmidt B. Epidemiology of bronchopulmonary dysplasia. Birth Defects Res A Clin Mol Teratol. (2014) 100:145–57. 10.1002/bdra.23235 24639412 PMC8604158

[35] Patel RM Kandefer S Walsh MC Bell EF Carlo WA Laptook AR. Eunice kennedy shriver national institute of child health and human development Neonatal research network. Causes and timing of death in extremely premature infants from 2000 through 2011. N Engl J Med. (2015) 372:331–40. 10.1056/NEJMoa1403489 25607427 PMC4349362

